# Polatuzumab‐bendamustine‐rituximab as bridge to CD19‐directed CAR T cells in mantle cell lymphoma refractory to ibrutinib and venetoclax

**DOI:** 10.1002/jha2.655

**Published:** 2023-04-10

**Authors:** Vincenzo Maria Perriello, Lorenza Falini, Loredana Ruggeri, Daniele Sorcini, Stelvio Ballanti, Leonardo Flenghi, Nicodemo Baffa, Piero Covarelli, Paolo Sportoletti, Antonio Pierini, Brunangelo Falini

**Affiliations:** ^1^ Hematology Section, Department of Medicine and Surgery, Center for Hemato‐Oncological Research (CREO) University of Perugia Perugia Italy; ^2^ Department of PET‐CT and Radiological and Laboratory Imaging Hospital Santa Maria della Misericordia Perugia Italy; ^3^ Section of Oncology Surgery, Department of Medicine and Surgery University of Perugia Perugia Italy

Classic mantle cell lymphoma (cMCL) is usually characterized by an aggressive clinical course [1, 2]. The Bruton's Tyrosine Kinase (BTK) inhibitors have significantly improved the outcome of relapsed/refractory (R/R) cMCL [3, 4]. However, unresponsive patients exhibit a poor prognosis, with a median overall survival (OS) of 6–10 months following salvage therapies [5]. In the ZUMA‐2 trial, CD19‐directed chimeric antigen receptor (CAR) T cells (brexucabtagene autoleucel) led to complete remission (CR) rate of about 60% in R/R cMCL (median follow‐up of 12.3 months) [6]. Median duration of response was 28.2 months whilst median progression‐free survival and OS were 25.8 and 46.6 months, respectively [7]. Similar results were also reported in the real‐world experience at a median follow‐up of 10.1 months [8].

Bridging approaches to CAR T cells have the goal to control lymphoma growth during manufacturing CAR T cells, especially in cases with progressive high tumour burden disease. Bridging therapy in cMCL have been so far limited to steroids, chemotherapy, radiotherapy, BTK inhibitors (ibrutinib, acalabrutinib or pirtobrutinib) [6] and venetoclax [8].

Here, we report a 47‐year‐old man with pleomorphic cMCL carrying a *TP53* mutation that, due to chemotherapy resistance and high tumour burden disease, was initially treated with ibrutinib + venotoclax as bridging to CAR T cell therapy. Because of lack of response, the patient underwent splenectomy followed by chemo‐immunotherapy with polatuzomab‐bendamustine‐rituximab [9], achieving an almost CR. This is the first time that such a CD79b directed regimen was successfully adopted as bridging to CAR T cells in cMCL. The biological and clinical significance of this finding are discussed.

A 47‐year‐old man was referred to our Institution for CAR T cell therapy because of cMCL refractory to chemotherapy and ibrutinib. A revision of the initial lymph node biopsy showed a cMCL, pleomorphic variant expressing CD5+ and cyclin D1, with high proliferative index (>50%). The bone marrow (BM) biopsy showed massive infiltration by pleomorphic MCL. Blood cell count was: WBC 10.5 × 10^9^/L with leukemic involvement, Hb 7.4 g/dL and platelets 12.0 × 10^9^/L. Fluorescence in situ hybridization (FISH) revealed a monoallelic deletion of *TP53* in about 50% of tumour cells. A positron emission tomography/computed tomography (PET/CT) showed a hypermetabolic uptake by multiple supra‐ and sub‐diaphragmatic lymph nodes (up to 5 cm in diameter), spleen (about 30 cm in its longest diameter) and BM. The patient was classified as high‐risk, according to the combination Mantle Cell Lymphoma International Prognostic Index [10].

Because of resistance to ibrutinib, mutational analysis of exon 15 of the *BTK* gene and exons 19‐20‐24 of the *PLC* gamma‐2 gene were performed but no mutations were detected. The patient was started on off‐label ibrutinib + venetoclax, as previously reported [11] that only led to a slight decrease in lymph nodes size, peripheral blood lymphocytosis and BM infiltration, without reduction of splenomegaly and no improvement of the severe anemia and thrombocytopenia (platelets 15,000/microl). The patient underwent an apheresis collection of lymphocytes.

Because of the unresponsive huge splenomegaly, we temporarily stopped ibrutinib + venetoclax to perform a splenectomy, following platelets infusion support. The spleen (3.9 Kg weight) was diffusely infiltrated by pleomorphic lymphoma cells (Figure 1A, B) strongly positive for CD5, cyclin D1, CD20, CD79a and CD79b (Figure 1C,E). Conversely, CD19 was expressed only in a percentage of tumour cells (Figure 1D), as also confirmed by flow cytometry (Figure 2A). The proliferation index was >50% (Figure 1F). Values of blood cell count returned to normal 1 week after spleen removal.

**FIGURE 1:**
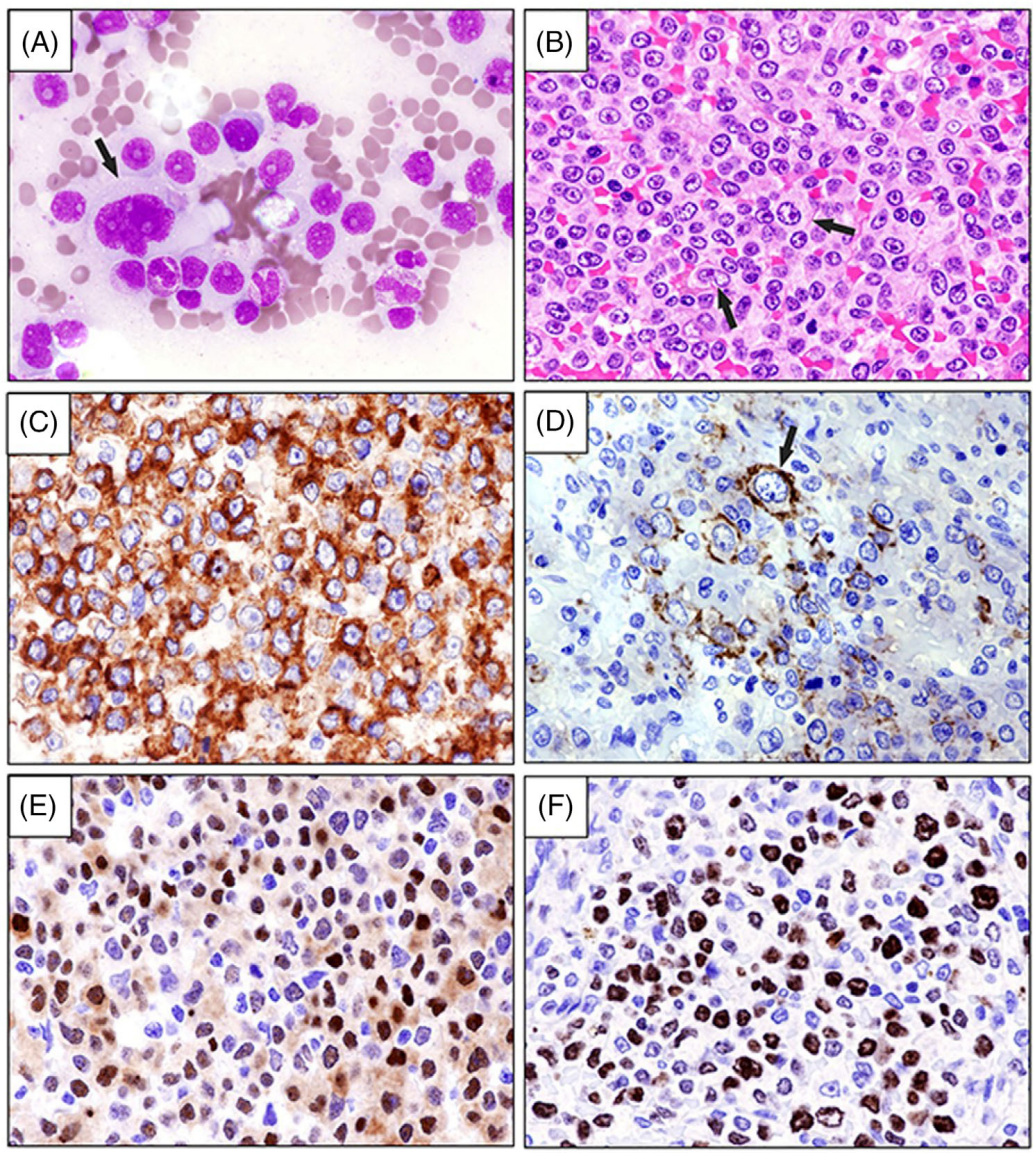
(A) Spleen imprint showing many mononucleated lymphoma cells with moderate‐large amount of cytoplasm and round nuclei with distinct single nucleolus. Occasional large pleomorphic cells are also seen (arrow) (May Grunwald Giemsa, x 400). (B) Diffuse spleen infiltration by medium‐ to large‐size (arrows) lymphoma cells showing evident nucleoli (hematoxylin‐eosin; x 400). (C–E) Tumour cells infiltrating the spleen strongly express CD79b (C, x 400) and are partially positive for CD19 (arrow) (D, x 400) and cyclin D1 (E, x 400). The percentage of Ki‐67 positive tumour cells is higher than 50% (F, x 400). (C–F) immunoperoxidase staining; hematoxylin counterstaining).

**FIGURE 2:**
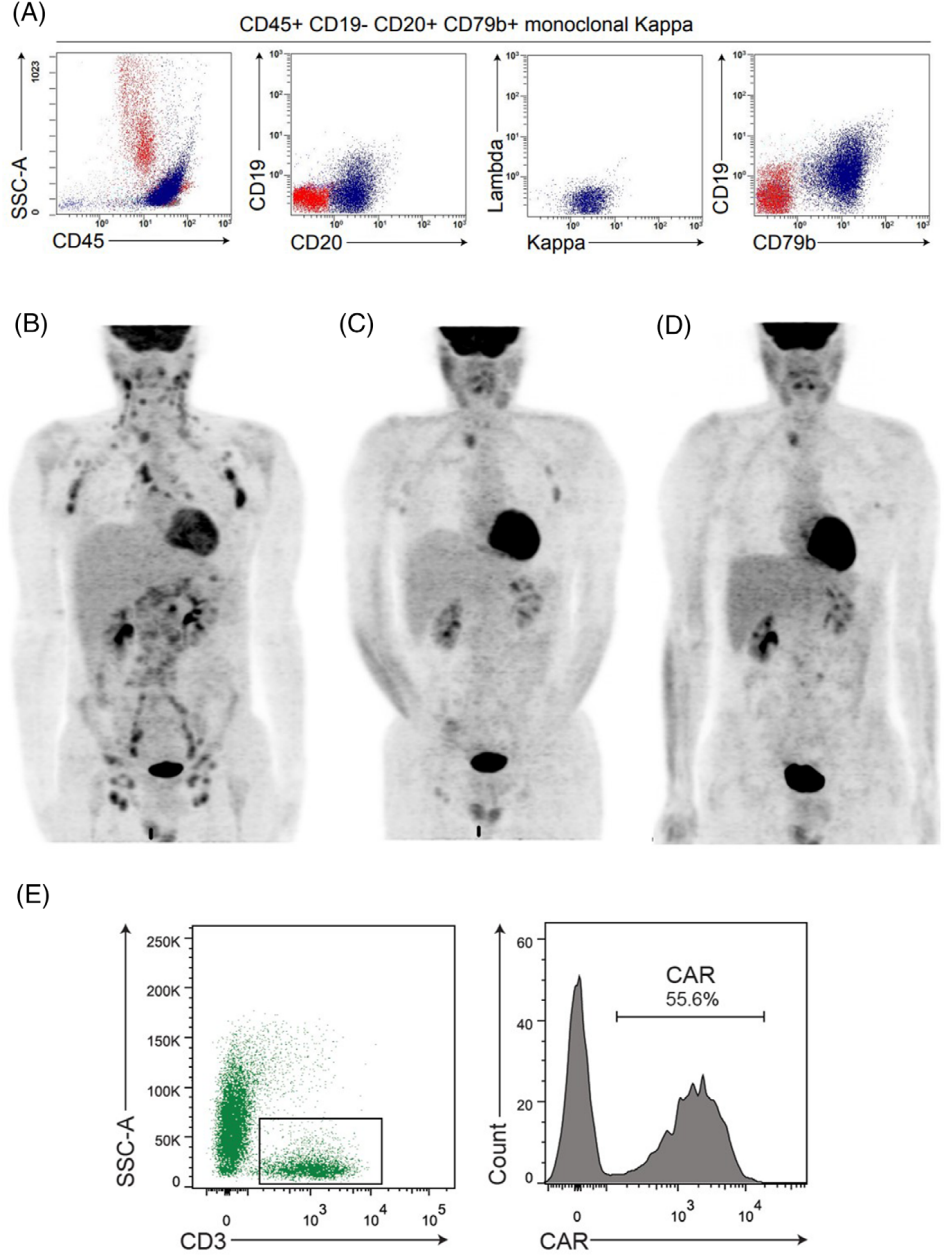
(A) Peripheral blood flow cytometry study before bridging therapy showed a kappa‐restricted monoclonal B cell population CD79B+ and CD19‐. (B–D) FDG‐positron emission tomography/computed tomography (PET/CT) coronal maximum intensity projection (MIP) image after ibrutinib+venetoclax and before Polatuzumab‐based regimen (B); FDG‐PET/CT after Polatuzumab‐based regimen showing almost complete metabolic complete response (C) reached 1‐month after CD19 CAR‐T cell therapy (D). (E) Flow cytometry plots showing CAR–T cell expansion in the CD3+ T cell subsets in the peripheral blood 14 days after CAR–T cell infusion. FDG, F‐fluorodeoxyglucose.

Because of the persistence of significant lymphadenopathy (Figure 2B) and the strong expression of CD79b, we used ‘off‐label’ polatuzomab‐bendamustine‐rituximab, as bridging to CAR T cells, achieving an almost CR after only one cycle (Figure 2C). The patient then underwent lymphocyte depletion with fludarabine/cyclophosphamide followed by brexucabtagene autoleucel infusion. A grade 1 cytokine release syndrome was observed. PET/CT scan performed 1 month after CAR T cells infusion showed a metabolic CR (Figure 2D) that was followed by relapse at month +3. Monitoring of CAR T cells expansion by flow cytometry showed high CAR T cell levels in peripheral blood, with values of 44/microliter (day +7), 310/microliter (day +14) and 18/microliter (day +14) (Figure 2E).

Polatuzumab‐bendamustine‐rituximab (Pola‐Benda‐R) has been approved for the treatment of refractory/relapsed (R/R) diffuse large B‐cell lymphomas [12], and it has also been used as bridging to CAR T cells in this setting [13]. This is the first time that a polatuzumab‐based regimen has been successful adopted as bridging to CAR T cells in cMCL resistant to ibrutinib and venetoclax. Polatuzumab is a CD79b‐binding monoclonal antibody conjugated to the anti‐mitotic agent monomethyl‐auristatin E [14, 15]. CD79b (and CD79a) serves as critical signaling components of the B‐cell receptor that promotes lymphoma survival [16, 17]. Following binding of polatuzumab to CD79b in B cells, monomethyl‐auristatin E is internalized and cleaved from its linker by lysosomal proteases leading to inhibition of microtubule polymerization, block of cell mitosis and induction of apoptosis [18, 19].

In the phase 1 trial exploring safety of polatuzumab as a single agent or combined with rituximab, an objective response was observed in almost all cMCL patients [20]. cMCL is one of non‐Hodgkin B‐cell lymphomas with highest expression of CD79b, in keeping with the finding that normal mantle zone B cells express more strongly CD79b than germinal center B cells (Supplementary Figure 1). Polatuzumab was the agent that likely played the major role in inducing remission in our patient, whilst an anti‐tumour effect of bendamustine appears unlikely since the patient was refractory to previous lines of chemotherapy and rituximab. Splenectomy may have contributed to improve the therapeutic effect of polatuzumab by reducing the large tumour mass and avoiding the sequestration of polatuzumab in the spleen (so‐called sink effect).

Another peculiar feature of our patient was the partial expression of CD19 by tumour cells in all body sites explored (peripheral blood, BM and spleen). In a previous study, 3/51 (5.8%) of MCL patients evaluable for response to CD19‐directed CAR T cells were CD19 negative [6]. Notably, all of them achieved a CR but no information was provided on the duration of the response. Our patient achieved an initial CR (month +1) that was followed by relapse (month +3), probably due the partial expression of CD19. The patient is now being considered for an allogeneic hematopoietic stem cell transplantation.

In conclusion, splenectomy plus polatuzumab‐based regimen was an effective bridging to CAR T cells therapy in our patient with high tumour burden R/R cMCL. This approach is worth to be explored in patients presenting with the same characteristics.

## AUTHOR CONTRIBUTIONS

VMP, LoFa, SB, LeFl, AP and PC managed the patient. BF carried out the pathological analysis. LR, DS and PS carried out flow cytometry analysis. NB performed PET‐CT. BF wrote the manuscript

## CONFLICT OF INTEREST STATEMENT

The authors declare they have no conflict of interest.

## FUNDING INFORMATION

The study was supported by Associazione Umbra per il trattamento delle Leucemie ed i Linfomi (AULL)

## ETHICS STATEMENT

A written informed consent was obtained from the patient.

## Supporting information

Figure S1 (A) Reactive lymph node with follicular hyperplasia. The CD79b molecule (brown) is more strongly expressed in the mantle zones than in the germinal centers (GCs) (asterisks) of the reactive B‐cell follicles (x100). (B) A higher magnification of a different field (x200). The asterisks indicate the GCs. (A and B) immunoperoxidase staining; hematoxylin counterstaining.

## Data Availability

Individual patient data will not be shared and data transfer Agreement will be required.
