# Supplementary figures and images for: Polatuzumab‐bendamustine‐rituximab as bridge to CD19‐directed CAR T cells in mantle cell lymphoma refractory to ibrutinib and venetoclax
Source: EJHaem. 2023 Apr 10;4(2):559–62. doi: 10.1002/jha2.655 (PMC10188502; doi:10.1002/jha2.655)

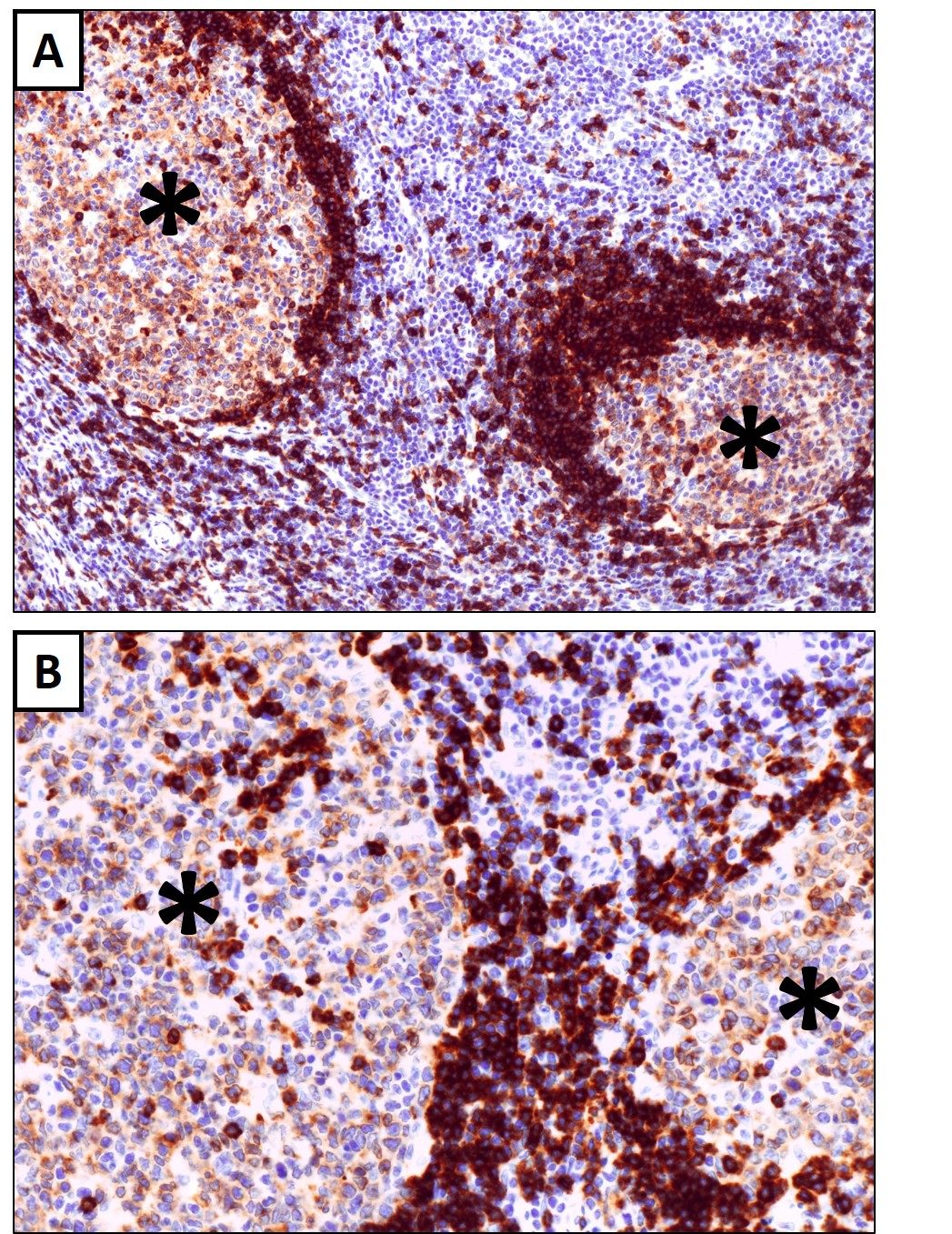

Supplement: Supplementary file 1 — Figure S1 (A) Reactive lymph node with follicular hyperplasia. The CD79b molecule (brown) is more strongly expressed in the mantle zones than in the germinal centers (GCs) (asterisks) of the reactive B‐cell follicles (x100). (B) A higher magnification of a different field (x200). The asterisks indicate the GCs. (A and B) immunoperoxidase staining; hematoxylin counterstaining. [file JHA2-4-559-s001.jpg]
